# Management of intervenable factors to reduce vascular complications in patients with internal carotid artery occlusion treated by non-emergency endovascular treatment

**DOI:** 10.3389/fneur.2024.1332940

**Published:** 2024-03-01

**Authors:** Guangyu Wu, Yuxin Nong, Shaorui Hong, Shuo Wang, Chengbo Dai, Chizhong He, Changmao Li, Tengyun Ma, Zhexian Yang, Bin Zhang, Yuyuan Gao, Guixian Ma

**Affiliations:** ^1^Department of Neurology, Guangzhou Key Laboratory of Diagnosis and Treatment for Neurodegenerative Diseases, Guangdong Neuroscience Institute, Guangdong Cardiovascular Institute, Guangdong Provincial People's Hospital, Guangdong Academy of Medical Sciences, Guangzhou, China; ^2^Department of Cardiology, Guangdong Provincial Key Laboratory of Coronary Heart Disease Prevention, Guangdong Cardiovascular Institute, Guangdong Provincial People's Hospital, Guangdong Academy of Medical Sciences, Guangzhou, China; ^3^Shantou University Medical College, Shantou, China; ^4^Department of Neurology, Guangdong Neuroscience Institute, Guangdong Provincial People's Hospital (Guangdong Academy of Medical Sciences), Southern Medical University, Guangzhou, Guangdong, China

**Keywords:** internal carotid artery occlusion, non-emergency endovascular treatment, vascular complications, glycosylated hemoglobin, non-neurology guide wires

## Abstract

**Objective:**

This study aims to identify risk factors for vascular complications during non-emergency endovascular treatment in patients with internal carotid artery occlusion (ICAO) and to propose potential interventions.

**Method:**

A retrospective analysis of 92 patients with ICAO who received non-emergency endovascular treatment in our center from 1 January 2018 to 31 June 2023, was conducted. The correlation between intraoperative vascular complications and potential risk factors was studied, and interaction analysis was performed.

**Results:**

Our findings revealed that the use of non-neurology guide wires to open vessels (adjusted OR: 4.1, 95%CI: 1.3–12.8; *p* = 0.014) and glycosylated hemoglobin (HbA1c) ≥ 6.5 mmol/L (adjusted OR: 3.2, 95%CI: 1.2–8.9; *p* = 0.023) was significantly associated with vascular complications in non-emergency endovascular treatment of ICAO patients. The restricted cubic spline (RCS) showed that the higher the HbA1c level, the higher the risk of vascular complications.

**Conclusion:**

The use of non-neurology guide wires for vessel opening during non-emergency endovascular treatment in patients with ICAO increases the risk of vascular complications. Preoperative assessment and management of HbA1c levels can reduce the incidence of intraoperative vascular complications.

## 1 Introduction

Chronic internal carotid artery occlusion (CICAO), defined as ICAO persisting for over 4 weeks. Atherosclerosis is the most common cause of internal carotid artery occlusion (ICAO), accounting for about 70% (1). It is more prevalent among the elderly population and men, and it is more likely to occur at the origin of internal carotid artery. Vascular endothelial damage can gradually progress to atherosclerosis and form plaques, resulting in the gradual formation of high stenosis of the vascular lumen, and finally evolve into CICAO. The majority of patients with CICAO are asymptomatic, thanks to collateral compensation, and exhibit a low stroke recurrence rate. Notably, only a small percentage of patients with CICAO experience embolism from thrombus or atherosclerotic plaque detachment at ICAO stumps, watershed infarction due to decreased cerebral perfusion pressure, or transient ischemic attack (TIA). Epidemiological data indicate that the incidence of symptomatic ICAO is approximately 6 cases per 100,000 individuals (2). Furthermore, patients with a history of TIA or mild strokes exhibit an annual recurrence risk of 5 to 6% (3). Notably, in the presence of hemodynamic disorders, the risk of stroke may be higher (4–6). The management of CICAO remains a subject of ongoing debate (7). Current therapeutic options include drug therapy, extracranial-to-intracranial bypass surgery, carotid endarterectomy (CEA), recanalization therapy, hybrid surgery. The efficacy of drug therapy is established, but challenges persist with CEA alone, which struggles to and intracranial long-segment occlusion. Hybrid surgery, though potentially promising, lacks comprehensive studies. Endovascular treatment emerges as a promising avenue, especially for patients long-segment occlusions (8, 9). Endovascular treatment of occlusive lesions poses unique challenges compared to arterial stenosis because of the involvement of longer segments, the frequent combination of underlying atherosclerosis and new thrombus, and making guidewire advancement more difficult (6, 10, 11). Furthermore, due to unclear true lumen path of vessels, soft or viscous organization of thrombus, and complications such as dissection, distal embolism, vascular rupture bleeding, pseudoaneurysm formation, fistula formation, and hyperperfusion syndrome (6, 8, 12), the incidence rate is considerably higher than stenotic lesions. Yet, the absence of prospective randomized trials evaluating the benefits of endovascular treatment beyond optimal drug therapy for symptomatic ICAO complicates the decision-making process. Rigorous screening by experienced neurologists may identify patients with CICAO who can benefit from vascular recanalization. Therefore, controlling intervenable factors in endovascular therapy holds the potential to diminish vascular complications and Increase the overall net benefit of endovascular therapy.

## 2 Methods

### 2.1 Study population

A total of 112 patients diagnosed with ICAO were admitted to our center for non-emergency endovascular treatment between 1 January 2018 and 31 June 2023. The Ethics Committee of Guangdong Provincial People’s Hospital approved the study (XJS2022-108-01). All patients were confirmed by computerized tomography angiography (CTA), magnetic resonance angiography (MRA), or digital subtraction angiography (DSA) that the occlusion plane accounted for 100% of the cross-sectional area of the vascular lumen. The inclusion criteria for endovascular treatment were as follows: patients with persistent or recurrent neurological deficits (stroke, TIA, and amaurosis fugax), ICAO indicated as responsible by magnetic resonance angiography (MRA) or CT angiography (CTA). Finally, patients were enrolled in this study after DSA verification in our center. Exclusions were applied to patients with ischemic strokes and a history of hemorrhagic disease within the past week, 8 patients with confirmed dissections based on imaging or DSA, 7 patients with a history of cancer radiotherapy, 2 patients with cardiogenic embolic type, and 3 patients with unexplained etiologies. Finally, 92 patients were selected with considered atherosclerotic lesions for endovascular treatment. The study analyzed demographic characteristics, atherosclerotic risk factors [Smoking history is defined according to the criteria in the American Adult Smoking Survey (13)], time to occlusion (7 to 730 days): time from the initial radiological diagnosis of occlusion by CTA/MRA or DSA to the commencement of endovascular treatment, the most recent laboratory parameters before surgery included HbA1c, triglycerides (TG), total cholesterol (TC), high-density lipoprotein cholesterol (HDL), and low-density lipoprotein cholesterol (LDL). Further details encompassed side of occlusion, presence of guidewire anchor points at the proximal stump of the occluded vessel suggested by DSA, the presence of blood flow compensation to the C4 segment of the internal carotid artery (ICA) and below [refer to ICA segmentation method proposed by Bouthillier et al. (14)], types of collateral pathways, the use of non-neurology dedicated guide wires, the utilization of embolic protection devices, the overall success of the recanalization procedure, the number of patients receiving percutaneous transluminal angioplasty (PTA), the number of patients receiving PTA and stenting (PTAS) and the Modified Rankin Scale (mRS) scores before the procedure.

### 2.2 Endovascular treatment

Prior to the procedure, all patients underwent magnetic resonance imaging (MRI) + MRA + diffusion-weighted image (DWI) and/or computed tomography (CT) and/or CTA and/or CT perfusion. Additionally, patients received rosuvastatin (10–20 mg/day) or atorvastatin (20–40 mg/day), dual antiplatelet therapy for at least 3 days preoperatively and were tested for platelet aggregation rates or clopidogrel drug metabolism gene testing (15–17). In cases indicating slow metabolism or unacceptable platelet aggregation rates, aspirin was switched to cilostazol, or clopidogrel was switched to ticagrelor (17, 18). Intravenous nimodipine injection was administered preoperatively to prevent vasospasm. For immediate neurological evaluation, all procedures were performed under local anesthesia. Heparin 3,000–5,000 U (70 U/kg) was intravenously injected at the beginning of coaxial catheter application, with additional doses of 1,000 U every hour thereafter. Conventional angiography was used to explore the occluded ICA and its collateral circulation, excluding pseudo-occlusion and near-occlusion based on literature criteria (19, 20). The technical challenge of intravascular recanalization is the safe passage of the guide wire through the occlusion segment. Given the long segmental nature of the occlusion and the softness of the intracranial blood vessels, the soft and flexible neuropathic microguide wire (Synchro, Transend) is preferred to carefully explore the occlusion surface and try to find anchorage points or weak points on the occlusion surface to break through the occlusion. When repeated attempts fail to pass the occlusion lesion, PILOT series, ASAHI Gaia series, Fielder XT series or runthrough, V18 Control Wire were selected to try again based on operator experience. Once the guidewire has passed through ICAO and the true lumen has been confirmed by angiography, the microguide wire was positioned distal to the middle cerebral artery (MCA) to act as a track for delivering a different device. A small-diameter angioplasty balloon was then used to expand from distal to proximal of the occlusion with appropriate pressure. If a suitable position is accessible, the cerebral protection device is used. In cases where transportation of the protection device to the appropriate position is challenging due to technical difficulties, such as vessel curvature, or if the occluded segment is excessively long, the use of the protection device is discontinued. Depending on intraoperative vascular lumen conditions, vessel diameter measured during the operation and at the discretion of the interventionalist, either PTA alone or further PTAS is deemed acceptable. Intraoperatively, if patients exhibited irritability or complained of pain affecting the interventionalist’s operation during balloon dilatation or stent placement, low-dose midazolam injection and sufentanil citrate injection were intravenously administered after excluding vascular complications. Occasionally, tirofiban hydrochloride and sodium chloride injection might be temporarily administered to patients with a high thrombus burden and repeated thrombosis attributed to vascular endothelial damage. Subsequently, a reassessment of intracranial angiography was conducted to evaluate both ipsilateral and contralateral perfusion. Recanalization was defined as the restoration of vessels with forward flow graded as mTICI2a or higher (21). CT scans immediately or MRI + DWI were performed postoperatively in some patients based on operator experience.

### 2.3 Observations and outcomes

The primary observational measures in this study encompassed all vascular complications occurring during endovascular treatment, including dissection, thrombosis, distal embolism, vascular rupture, and arteriovenous fistula. Secondary measures included the assessment of collateral circulation patency and mRS scores at discharge.

The primary outcome aimed at identifying risk factors for vascular complications during endovascular treatment, with separate analyses testing interaction effects of included variables on these risk factors. The secondary outcome focused on determining the proportion of patients with a mRS score of ≤2 at discharge.

### 2.4 Statistical analysis

The data distributions of each covariate between the group without vascular complications and the group with vascular complications were compared. Continuous variables with a normal distribution were evaluated using t-tests, while those with non-normal distributions were evaluated with the Mann–Whitney U test. Categorical data were analyzed using the χ2 test or Fisher’s exact test. Among the 92 patients in the study, HbA1c levels were missing for 4 (4%) patients and all lipid levels for 2 (2%) patients. To address these missing data, regression estimation was employed. Subsequently, the covariates were analyzed. These covariates were independent of each other, and categorical variables were classified comprehensively and in a mutually exclusive manner. Linear regression was used to test for multicollinearity among covariates. Following these preliminary steps, all covariates were analyzed by univariate Logistic regression. Covariates with a value of *p* < 0.2 (22) in univariate Logistic regression analysis, along with factors theoretically likely to impact vascular complications, were included in the multivariate Logistic regression analysis. The presence of any interactions between the results obtained from the multi-factor logistic regression analysis and other independent variables was explored. Furthermore, the relationship between HbA1c levels and intraoperative vascular complications was investigated using RCS. All statistical analyses were conducted using SPSS statistical software (v26.0) and plotted using GraphPad Prism v10.0.2 and R.^1^ *p*-values less than 0.05 were considered statistically significant.

## 3 Results

**Figure 1:**
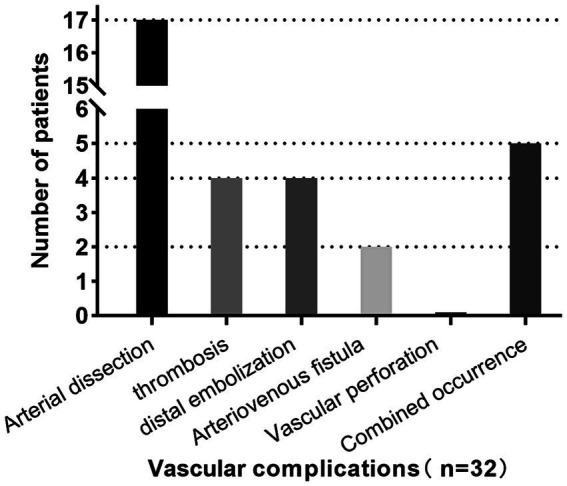
Vascular complications occurred in 32 patients (33%), including arterial dissection in 17 patients (53%), thrombosis *in situ* and distal embolism in 4 patients (13%) respectively, arteriovenous fistula in 2 patients (6%), more than one vascular complication in 5 patients (16%). Neurologic localization symptoms occurred in 2 patients (6%), both of which were caused by distal embolization. No vascular rupture occurred.

**Figure 2:**
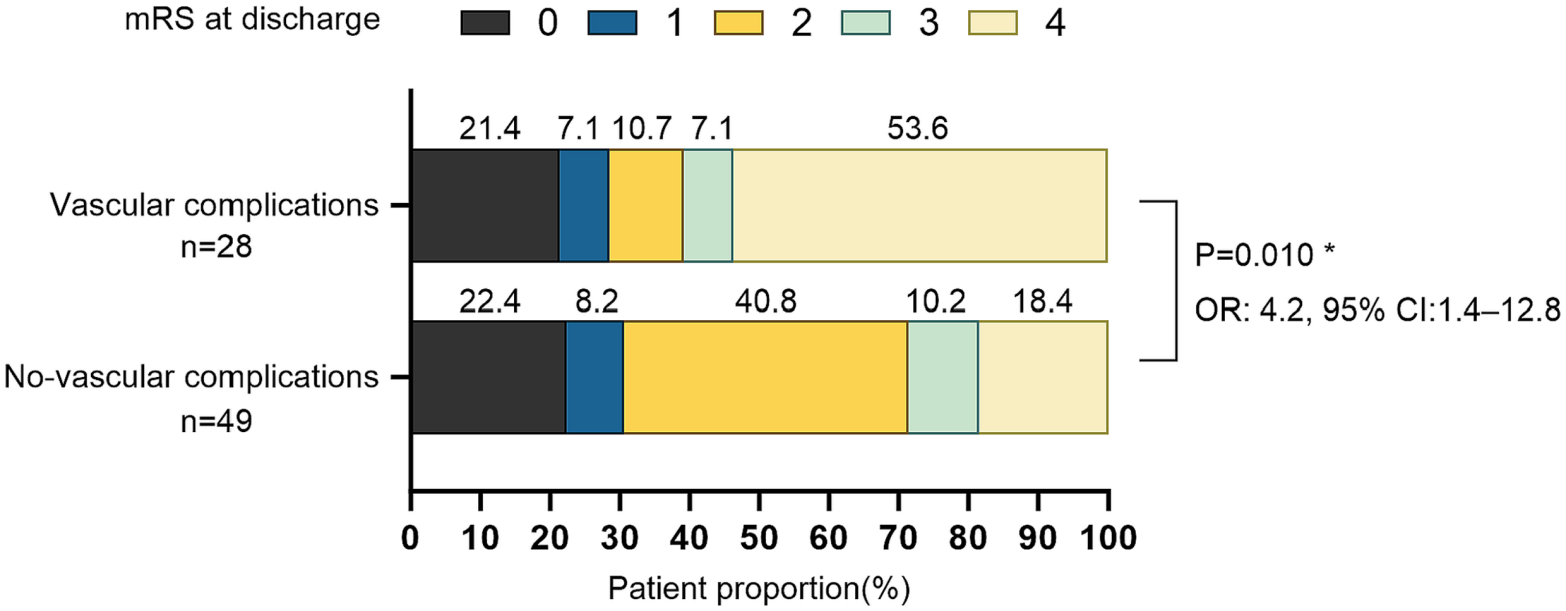
Distribution of mRS scores in patients with revascularization at discharge (*N* = 77). *Odds ratios (ORs) for the mRS ≥ 3, adjust with smoking, hypertension, diabetes, LDL-C, occlusion time.

Successful endovascular recanalization was achieved in 77 out of 92 patients (84%), of which 59 patients (84%) underwent PTA + PTAS. Conditions of other intracranial and extracranial arteries are detailed in Supplementary Table 1. Among the cohort, 32 patients (33%) experienced vascular complications during the endovascular treatment, with only two presenting neurological localization signs. Specifically, one case exhibited Broca aphasia, and another displayed a reduction in left limb muscle strength from grade 3 preoperatively to grade 2 postoperatively, accompanied by a change in consciousness from wakefulness to lethargy. 38 CT scans were performed immediately postoperatively, revealing no instances of bleeding. Demographic characteristics, laboratory parameters, the distribution of devices used during therapy and mRS scores before the procedure are summarized in Table 1. No intergroup differences were seen in the type of collateral pathways (Table 1). Intraoperative, arterial dissection occurred in 17 patients (53%), thrombosis *in situ* and distal embolism in 4 patients (13%) respectively, arteriovenous fistula in 2 patients (6%), multiple vascular complications in 5 patients (16%; Figure 1). An increased proportion of patients with recanalization who experienced intraoperative vascular complications had an mRS ≥ 3 at discharge (adjusted OR, 4.2 [95%CI: 1.4–12.8]; *p* = 0.010; Figure 2).

**Table 1:** Demographic and clinical characteristics of the cases included in the study.

Variables	During the endovascular treatment		
	No-vascular complications (*n* = 60)	Vascular complications (*n* = 32)	Value of *p*
Age, years	64 (8.8)	62 (8.0)	0.401
Gender, *n* (%)			>0.999
Male	52 (87)	28 (87.5)	
Female	8 (13)	4 (12.5)	
Medical history, *n* (%)			
Hypertension			0.112
No	25 (42)	8 (25)	
Yes	35 (58)	24 (75)	
Type 2 diabetes			0.969
No	34 (57)	18 (56)	
Yes	26 (43)	14 (44)	
Smoking, *n* (%)			0.105
No	35 (58)	13 (41)	
Yes	25 (42)	19 (59)	
Imaging diagnosis to operation time, *n* (%)			0.894
≤4 weeks	31 (52)	17 (53)	
>4 weeks	29 (48)	15 (47)	
HbA1c, *n* (%)			0.065
<6.5 mmol/L	45 (75)	18 (56)	
≥6.5 mmol/L	15 (25)	14 (44)	
TG (mmol/L)	1.30 (0.93, 1.68)	1.11 (0.88, 1.79)	0.314
TC (mmol/L)	3.83 (3.33, 4.91)	3.71 (2.94, 4.92)	0.456
HDL (mmol/L)	0.93 (0.82, 1.09)	1.01 (0.83, 1.26)	0.348
LDL (mmol/L)	2.42 (2.00, 3.32)	2.22 (1.81, 3.04)	0.320
Side of blocked, *n* (%)			0.404
Left	26 (43)	11 (34)	
Right	34 (57)	21 (66)	
ICA stump morphology, *n* (%)			0.193
No anchor point	21 (35)	7 (22)	
Anchor point	39 (65)	25 (78)	
Compensate to the C4 segment of the internal carotid artery and below, *n* (%)			0.058
No	35 (58)	25 (78)	
Yes	25 (42)	7 (22)	
Types of collateral pathways, *n* (%)			0.159
None	3 (5)	1 (3)	
AcoA	5 (8)	7 (22)	
ACoA + PCoA	5 (8)	6 (19)	
ACoA + E-I	17 (28)	5 (16)	
ACoA + PCoA + E-I	13 (22)	2 (6)	
PCoA	3 (5)	3 (9)	
PCoA+E-I	6 (10)	4 (12)	
E-I	8 (13)	4 (12)	
Additional use of non-neurology guide wires, *n* (%)			0.173
No	37 (62)	15 (47)	
Yes	23 (38)	17 (53)	
Use of embolic protection devices, *n* (%)			0.158
No	45 (75)	28 (87.5)	
Yes	15 (25)	4 (12.5)	
Successful endovascular revascularization, *n* (%)			0.471
No	11 (18)	4 (12.5)	
Yes	49 (82)	28 (87.5)	
*N* of PTA	11 (18)	7 (22)	0.784
*N* of PTA + PTAS	38 (63)	21 (66)	>0.999
mRS scores before the procedure, *n* (%)			0.116
0	11 (18)	5 (16)	
1	9 (15)	5 (16)	
2	23 (38)	6 (19)	
3	7 (12)	3 (9)	
4	10 (17)	13 (41)	

AcoA, anterior communicating artery; PCoA, posterior communicating artery; E-I, extracranial-to-intracranial collaterals.

Notably, 44 patients (48%) with CICAO. Additionally, 29 patients (31.5%) had HbA1c levels outside the normal range. Given the challenging vascular path and poor vascular lumen conditions, 73 patients (79%) did not utilize embolic protection devices during therapy. Peripheral or coronary guide wires were used intraoperatively in 40 patients (43.5%) following unsuccessful attempts with Synchro and Transend guide wires due to the difficulty of advancing the guidewire through the occluded segment. Univariate logistic regression analysis of independent variables showed results presented in Table 2. Taking into account both the univariate analysis and clinical theory, adjusting for HbA1c levels, occlusion stump morphology, use of non-neurology guide wires, intraoperative use of embolic protection devices, and successful recanalization of occluded vessels, multivariate logistic regression analysis demonstrated that preoperative HbA1c ≥6.5 (OR: 3.2, 95%CI: 1.2–8.9; *p* = 0.023), and the use of non-neurology guide wires (OR: 4.1, 95%CI: 1.3–12.8; *p* = 0.014) were significantly associated with vascular complications during non-emergency endovascular treatment in patients with ICAO (Table 3).

**Table 2:** Effects of risk factors on vascular complications in non-emergency endovascular treatment of internal carotid artery occlusion by univariate analysis.

Variables	Total	Odds ratio (95% CI)	Value of *p*
Medical history, *n* (%)			0.116
Hypertension	59 (64)	2.14 (0.83, 5.54)	0.116
Type 2 diabetes	40 (44)	1.02 (0.43, 2.42)	0.969
Smoking, *n* (%)	44 (48)	2.05 (0.86, 4.90)	0.108
Imaging diagnosis to operation time, *n* (%)			0.894
≤4 weeks	48 (52)	1	
>4 weeks	44 (48)	0.94 (0.40, 2.23)	
HbA1c, *n* (%)			0.068
<6.5 mmol/L	63 (68.5)	1	
≥6.5 mmol/L	29 (31.5)	2.33 (0.94, 5.80)	
TG (mmol/L)	1.25 (0.91, 1.74)	0.72 (0.38, 1.38)	0.324
TC (mmol/L)	3.77 (3.21, 4.91)	0.87 (0.59, 1.26)	0.452
HDL (mmol/L)	0.96 (0.82, 1.12)	2.52 (0.37, 17.23)	0.345
LDL (mmol/L)	2.40 (1.93, 3.23)	0.77 (0.46, 1.29)	0.317
Side of blocked, *n* (%)			0.405
Left	37 (40)	1	
Right	55 (60)	1.46 (0.60, 3.56)	
ICA stump morphology, *n* (%)			0.196
No Anchor point	28 (30)	1	
Anchor point	64 (70)	1.92 (0.71, 5.19)	
Compensate to the C4 segment of the internal carotid artery and below, *n* (%)			0.062
No	60 (65)	1	
Yes	32 (35)	0.39 (0.15, 1.05)	
Additional use of non-neurology guide wires, *n* (%)			0.175
No	52 (56.5)	1	
Yes	40 (43.5)	1.82 (0.77, 4.34)	
Use of embolic protection devices, *n* (%)			0.166
No	73 (79)	1	
Yes	19 (21)	0.343 (0.13, 1.42)	
Successful endovascular revascularization, *n* (%)			0.473
No	15 (16)	1	
Yes	77 (84)	1.57 (0.46, 5.40)	

**Table 3:** Multivariate logistic regression model for risk factors associated with vascular complications in non-emergency endovascular treatment of internal carotid artery occlusion.

Variables	Adjusted OR (95% CI)	Value of *p*
HbA1c ≥ 6.5 mmol/L	3.2 (1.2, 8.9)	0.023
Guide wires Anchor point	3.1 (0.9, 10.5)	0.065
Additional use of non-neurology guide wires	4.1 (1.3, 12.8)	0.014
Use of embolic protection devices	0.4 (0.1, 1.3)	0.127
Successful endovascular revascularization	2.8 (0.7, 11.7)	0.163

Furthermore, we used RCS to flexibly model and visualize the correlation between HbA1c levels and vascular complications during non-emergent endovascular therapy in patients with ICAO, and the analysis suggests that the risk of vascular complications increases with increasing HbA1c levels (Supplementary Figure 1).

## 4 Discussion

Our study revealed an 84% recanalization rate of ICAO endovascular treatment at our center, consistent with previous literature (8, 9). Patients with ICAO undergoing emergency endovascular treatment are typically in urgent and critical condition, most of the existing studies are aimed at expanding the indications for surgery and giving patients greater benefits. While vascular events are closely related to perioperative blood pressure control, there are currently no specific guideline recommendations in this regard. Our center manages the postoperative blood pressure levels of patients according to the DAWN Trial (DWI or CTP Assessment With Clinical Mismatch in the Triage of Wake Up and Late Presenting Strokes Undergoing Neurointervention With Trevo) in combination with the patient’s baseline blood pressure and vascular patency (23), and we have not performed an analysis of postoperative vascular complications for these reasons.

We only retrospectively analyzed vascular complications during endovascular treatment and did not include radiographic follow-up of patients in this study, thus missing the proportion of delayed pseudoaneurysm formation due to intimal injury, which requires further follow-up to supplement the study. From a pathological standpoint, ICA dissection, ICAO after cancer radiotherapy, and cardiogenic embolism differ in terms of vascular endothelial status and injury mechanisms when compared to atherosclerosis (24–27). Therefore, to minimize the impact on the study, we excluded patients with non-large atherosclerotic ICAO from our analysis. Furthermore, Collateral circulation in patients with ICAO includes connections through the circle of Willis, connections to the leptomeningeal, and extracranial-intracranial collateral circulation. These collateral circuits compensate for reduced blood flow, and the adequacy of compensation leads to large differences in clinical performance (28). Our study did not observe further benefits (impact on intraoperative vascular complications) from these collateral circulations. However, the results must be interpreted with caution due to the small sample size of the subgroups for each collateral type.

Another critical factor to consider is the occlusion time. Previous studies have shown that longer occlusion times are associated with lower success rates of patency. The accurate determination of occlusion time can be challenging. Here, we defined occlusion time as the time from the diagnosis of ICAO based on imaging or DSA to the day when endovascular treatment starts. With the extension of time following vascular occlusion, thrombosis may continue, the vascular wall may collapse due to reduced intravascular blood flow, and the occluded segment can undergo fibrosis. These factors collectively make it increasingly challenging to identify the true lumen and raise the likelihood of vascular damage during intraoperative guide wire passage (8, 9, 12). Thus, occlusion time was also adjusted in the analysis. After adjusting for these potential factors, we observed significant associations between elevated HbA1c levels and the use of non-neurology guide wires with vascular complications during non-emergency endovascular treatment in patients with ICAO. Elevated blood glucose levels can increase the risk of vascular complications through multiple biological mechanisms, including coagulation and fibrinolytic dysfunction (29, 30), as well as endothelial dysfunction (31). Due to the popularity of medical insurance and the improvement of national health awareness, relatively few patients with HbA1c levels above the normal range in our study, coupled with the fact that patients with ICAO are mostly asymptomatic and only a few patients require endovascular treatment, might explain the wide confidence interval in the 95% CI of the OR value in Figure 2. Consequently, multicenter and large-scale studies are needed to verify and support this conclusion.

Compared with Synchro and Transend, other commonly used guide wires in our center, such as the PILOT series, ASAHI Gaia series, and Fielder XT series, are notably stiffer. While they have a stronger ability to break through chronic total occlusion lesions, it’s essential to recognize that guide wires slide forward within the vascular lumen, the rigidity of these guide wires may increase the risk of damage to vascular intima and collagen exposure beneath the vascular intima during penetration, resulting in vascular dissection, rupture, or further coagulation initiation to aggravate thrombus load. These complications may prolong the duration of guide wires and catheters remaining in the vessel and lead to increased stent placement.

Nonetheless, the possibility of selection bias and sample size from a single center may influence the generalizability of our findings. Among the 32 patients who experienced vascular complications during endovascular treatment in our study, only 2 had signs of neurological localization: Broca aphasia occurred in 1 case, left limb muscle strength changed from grade 3 before operation to grade 2 after operation, and consciousness changed from consciousness to lethargy. These observations indicate that most patients with severe perfusion defects were asymptomatic, even if they display vascular complications. However, increasing the remedial measures may increase the risk of complications, which is unfavorable for the patients.

## 5 Conclusion

Caution should be exercised when using non-neurology guide wires for vessel opening during non-emergency endovascular treatment in patients with ICAO, as this may increase the risk of intraoperative vascular complications. Additionally, it is advisable to control HbA1c levels preoperatively to mitigate the risk of intraoperative vascular complications.

## Data availability statement

The raw data supporting the conclusions of this article will be made available by the authors, without undue reservation.

## Ethics statement

The Ethics Committee of Guangdong Provincial People’s Hospital approved the study (XJS2022-108-01). The studies were conducted in accordance with the local legislation and institutional requirements. Written informed consent for participation was not required from the participants or the participants’ legal guardians/next of kin in accordance with the national legislation and institutional requirements.

## Author contributions

GW: Conceptualization, Data curation, Methodology, Software, Validation, Writing – original draft, Investigation. YN: Conceptualization, Data curation, Methodology, Software, Validation. SH: Investigation, Validation. CD: Investigation, Validation. CH: Investigation, Validation. CL: Investigation, Validation. TM: Investigation, Validation. ZY: Investigation, Validation. BZ: Investigation, Validation. YG: Investigation, Validation. GM: Conceptualization, Data curation, Funding acquisition, Investigation, Methodology, Software, Validation, Writing – review & editing.

## Footnotes

^1^www.r-project.org/
