# Supplementary material for: Management of intervenable factors to reduce vascular complications in patients with internal carotid artery occlusion treated by non-emergency endovascular treatment
Source: Front Neurol. 2024 Mar 1;15:1332940. doi: 10.3389/fneur.2024.1332940 (PMC10940403; doi:10.3389/fneur.2024.1332940)
Supplement: Supplementary file 1 [file Presentation_1.pdf]

Supplementary Material

Supplementary Figures

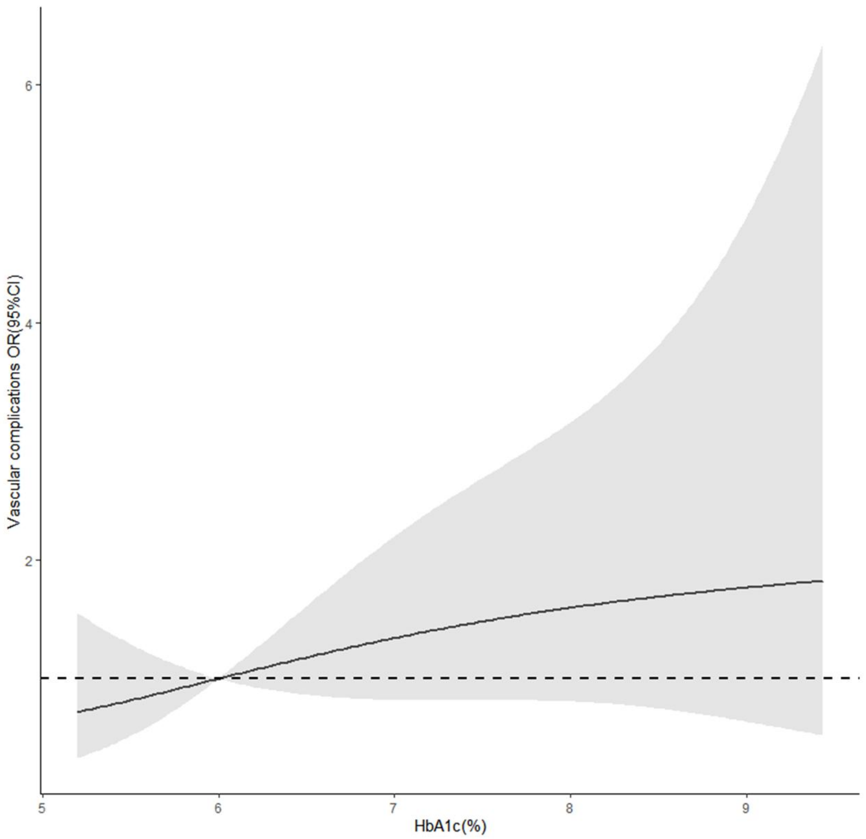

Supplementary Figure 1. Supplementary Figure 1. A nonlinear relationship between the HbA1c level and risk of vascular complications was observed. The risk of vascular complications increases with increasing HbA1c levels.

Supplementary Tables

	Total	N of endovascular treatments
Contralateral ICA≥70% stenosis	9	9
Contralateral ICAO	4	0
Ipsilateral MCA/ACA≥70% stenosis or occlusion	5	0
Contralateral MCA/ACA≥70% stenosis or occlusion	8	0
Veretbral arteries≥70% stenosis	16	9[#]
Veretbral arteries occlusion	5	0
Basilar artery ≥70% stenosis	2	0

[#] We restricted endovascular therapy to the dominant vertebral artery.

Supplementary Table 1. Conditions of intracranial and extracranial arteries other than ICAO.
